# TeleKap-Supported Intracerebral Haemorrhage Care in Slovenia: Organisational Framework, Potential Benefits, and Evidence Gaps

**DOI:** 10.3390/jcm15166200

**Published:** 2026-08-11

**Authors:** Tomaz Velnar, Katarina Salobir, Bruno Splavski, Borut Prestor, Senta Frol, Ulf Jensen-Kondering, Matija Zupan

**Affiliations:** 1 Department of Neurosurgery, University Medical Centre, 1000 Ljubljana, Slovenia; katarina.salobir@gmail.com (K.S.); borut.prestor@kclj.si (B.P.); 2 Medical Faculty, University of Ljubljana, 1000 Ljubljana, Slovenia; senta.frol@kclj.si (S.F.); matija.zupan@kclj.si (M.Z.); 3 Alma Mater Europaea University, 2000 Maribor, Slovenia; 4 Department of Neurosurgery, Dubrovnik General Hospital, 20000 Dubrovnik, Croatia; splavuno@gmail.com; 5 University of Applied Health Sciences, 10000 Zagreb, Croatia; 6 Department of Vascular Neurology, University Medical Centre, 1000 Ljubljana, Slovenia; 7 Department of Neuroradiology, University of Schleswig-Holstein, Campus Lübeck, 23552 Lübeck, Germany; ulf.jensen-kondering@uksh.de

**Keywords:** TeleKap (TeleStroke), telemedicine, Slovenia, intracerebral haemorrhage, neurosurgery

## Abstract

**Background:** Intracerebral haemorrhage (ICH) requires rapid multidisciplinary assessment, yet access to neurological and neurosurgical expertise may be uneven. Although telemedicine is established in acute ischaemic stroke (AIS), evidence regarding its role in ICH remains limited. This review describes the organisational framework of the Slovenian TeleKap network and examines its potential application to ICH care. **Methods:** PubMed/MEDLINE, Scopus, and Google Scholar were searched for literature concerning telemedicine, telestroke, ICH, neurosurgery, acute stroke care, and the Slovenian TeleKap network. Relevant guidelines, reviews, clinical studies, and publications describing telemedicine implementation were narratively synthesised, with emphasis on organisational workflow and neurosurgical consultation. **Results:** TeleKap connects regional hospitals with tertiary neurological and neurosurgical centres through audiovisual consultation and remote access to clinical and imaging data. In ICH, it provides an organisational mechanism for specialist input into assessment, triage, transfer decisions, preoperative planning, local management, and continuity of care. These functions constitute organisational capabilities rather than clinically validated benefits. They may support more standardised workflows and multidisciplinary coordination; however, no TeleKap-specific ICH registry, consecutive cohort, or comparative study has demonstrated shorter treatment times, more appropriate transfers, improved resource utilisation, or better clinical outcomes. Published evidence of telestroke effectiveness relates predominantly to AIS and cannot be extrapolated directly to ICH. **Conclusions:** TeleKap represents a national implementation of established telemedicine principles within the Slovenian healthcare system, but its clinical effectiveness, cost-effectiveness, and transferability remain to be established. Further development should prioritise registry-based quality monitoring, standardised protocols, and interoperability, with locally validated AI-supported imaging considered as a subsequent step.

## 1. Introduction

Intracerebral haemorrhage (ICH) is a devastating form of stroke, accounting for approximately 10–15% of all cerebrovascular events yet contributing disproportionately to mortality and long-term disability worldwide. Despite advances in neurocritical care, the prognosis of ICH remains poor, with reported 30-day mortality rates ranging from 30% to 50%, and only a minority of survivors achieving functional independence [1,2]. Early neurological deterioration is common, often occurring within the first hours after symptom onset, underscoring the critical importance of rapid diagnosis, prompt risk stratification, and timely initiation of appropriate management strategies [3].

Recent linked nationwide data provide the clearest contemporary overview of ischaemic stroke care in Slovenia. Among 16,839 patients admitted during 2015–2022, the crude admission rate decreased modestly from 105 to 99 per 100,000 inhabitants, while intravenous thrombolysis increased from 6.2% to 15.0% and mechanical thrombectomy from 5.4% to 9.5%, demonstrating substantial expansion of reperfusion treatment [4].

By contrast, recent Slovenian publications provide considerably less information on spontaneous intracerebral haemorrhage and subarachnoid haemorrhage, both of which were excluded from this nationwide analysis. This is an important limitation because haemorrhagic stroke remains associated with disproportionately high mortality and disability despite its lower incidence [5,6]. The absence of a comprehensive national stroke registry particularly restricts assessment of haemorrhagic stroke incidence, anticoagulant-associated bleeding, reversal treatment, access to neurosurgical or endovascular care, and functional outcomes. Given its nationwide reach, TeleKap could provide infrastructure for prospective surveillance and more standardised specialist management of both ischaemic and haemorrhagic stroke, rather than functioning solely as a pathway for reperfusion selection.

Management of ICH is complex and requires a multidisciplinary approach involving neurologists, neurosurgeons, radiologists, and intensive care specialists, while clinical decision-making is highly time-sensitive [7,8]. However, access to specialized neurosurgical expertise is not uniformly available, particularly in smaller or regional hospitals, which can lead to delays in decision-making and poorer patient outcomes. In this setting, telemedicine has arisen as a substantial device in the healthcare delivery system. It is described as the use of information and communication technologies to ensure remote clinical services, allowing real-time collaboration among healthcare providers across different locations [7,8,9]. During the last two decades, telemedicine has been integrated into acute stroke care, giving rise to the concept of “telestroke” networks that have been shown to reduce treatment delays, increase the use of reperfusion therapies, and improve clinical outcomes in patients with acute ischaemic stroke (AIS) [10,11]. The same infrastructure may also support haemorrhagic-stroke assessment, but benefits demonstrated in acute ischaemic stroke (AIS) should not be assumed to apply directly to ICH.

The anatomical location of intracerebral haemorrhage has important implications for its underlying aetiology, clinical presentation, prognosis, and management [7,8]. Deep subcortical haemorrhages, typically involving the basal ganglia, thalamus, brainstem, or cerebellum, are most commonly associated with chronic hypertensive small-vessel disease. In contrast, lobar haemorrhages more frequently result from non-hypertensive causes, particularly cerebral amyloid angiopathy, vascular malformations, tumours, coagulopathies, or other structural vascular lesions. Recent evidence has demonstrated that patients with lobar ICH exhibit a distinct clinical profile and experience a more severe early prognosis than those with deep subcortical haemorrhage, including higher in-hospital mortality (26.7% vs. 16.5%) and differences in presenting neurological symptoms. These findings highlight the importance of considering haemorrhage location during early specialist assessment and patient triage, making rapid telemedicine-supported evaluation particularly valuable in optimizing treatment decisions [9,10,12].

Although intracerebral haemorrhage is generally associated with high mortality and severe disability, its clinical presentation and prognosis are heterogeneous. A distinct subgroup comprises haemorrhagic lacunar stroke, characterized by small deep intracerebral haemorrhages that present clinically with classic lacunar syndromes. This subtype accounts for approximately 7.4% of intracerebral haemorrhages and is generally associated with a more favourable early prognosis than larger intracerebral haemorrhages, with no in-hospital mortality reported in the original clinical series and complete symptom resolution at hospital discharge in approximately 22.8% of patients. These observations illustrate the marked heterogeneity of ICH and emphasize the importance of accurate early diagnosis and individualized management strategies [13]. The clinical heterogeneity of intracerebral haemorrhage should also be recognized when considering telemedicine-supported management. While large lobar haemorrhages frequently require urgent neurosurgical assessment and transfer, smaller deep haemorrhages presenting as haemorrhagic lacunar stroke often follow a considerably more favourable clinical course and are usually managed conservatively [10,11,13]. In this context, TeleKap contributes by enabling rapid specialist evaluation, allowing patients with favourable imaging and clinical characteristics to remain safely in regional hospitals while identifying those requiring escalation of care [13].

In patients with ICH, telestroke systems may facilitate early imaging review, identification of high-risk features, and referral to specialised centres when neurosurgical intervention is considered [14]. Moreover, they support decision-making in complex clinical scenarios, such as the management of anticoagulated patients or those with significant comorbidities.

In Slovenia, the TeleKap system represents a nationwide implementation of a telestroke network designed to optimize the management of acute stroke patients. Introduced in 2014, TeleKap connects all regional hospitals with tertiary neurological and neurosurgical centres, enabling continuous access to specialist consultation. While the primary focus of TeleKap was initially on improving access to thrombolytic therapy in AIS, its role has evolved significantly over time. The system is also used operationally for neurosurgical emergencies, including ICH. This use reflects the clinical rationale for obtaining timely neurosurgical input in selected patients, especially those with large hematomas, mass effect, or hydrocephalus requiring urgent surgical intervention.

Because evidence regarding telestroke-supported pathways for spontaneous ICH remains less developed than that for AIS, this review examines the Slovenian TeleKap network with emphasis on patient triage, neurosurgical decision-making, interhospital coordination, and multidisciplinary care. Hub-and-spoke telestroke systems, remote image review, specialist-supported transfer decisions, and teleneurosurgical consultation have already been described in several settings [15,16,17,18,19,20,21,22]. Accordingly, we do not propose that either the organisational model or its application to neurosurgical emergencies is unique. Rather, TeleKap is presented as a country-specific example of how established telemedicine principles have been incorporated into a nationwide stroke infrastructure. This conceptual and practice-based description does not establish that TeleKap reduces delays or improves clinical outcomes in ICH.

## 2. Methods

This article was conducted as a narrative review aimed at providing an overview of the role of the Slovenian TeleKap network in the management of spontaneous intracerebral haemorrhage. A literature search was performed using the PubMed/MEDLINE, Scopus, and Google Scholar databases to identify relevant publications on telemedicine, telestroke systems, intracerebral haemorrhage, neurosurgical management, and acute stroke care. The search included combinations of keywords such as telemedicine, telestroke, TeleKap, intracerebral haemorrhage, intracerebral haemorrhage, stroke network, neurosurgery, telehealth, and Slovenia.

Priority was given to international clinical guidelines, systematic reviews, randomized clinical trials, observational studies, and key publications describing telemedicine implementation in stroke systems of care. Additional references were identified through manual screening of the reference lists of relevant articles. Publications written in English and considered directly relevant to the objectives of this review were included, preferably from 2000 to 2026. Articles not related to telemedicine or acute intracerebral haemorrhage management, conference abstracts without sufficient methodological in-formation, and duplicate publications were excluded.

As this is a narrative review, no formal systematic review methodology, protocol registration, or formal quality assessment of individual studies was performed. The selected literature was synthesized narratively, with particular emphasis on the organization, clinical workflow, and neurosurgical application of the Slovenian TeleKap system.

## 3. Overview of the TeleKap System in Slovenia

**Figure 1:**
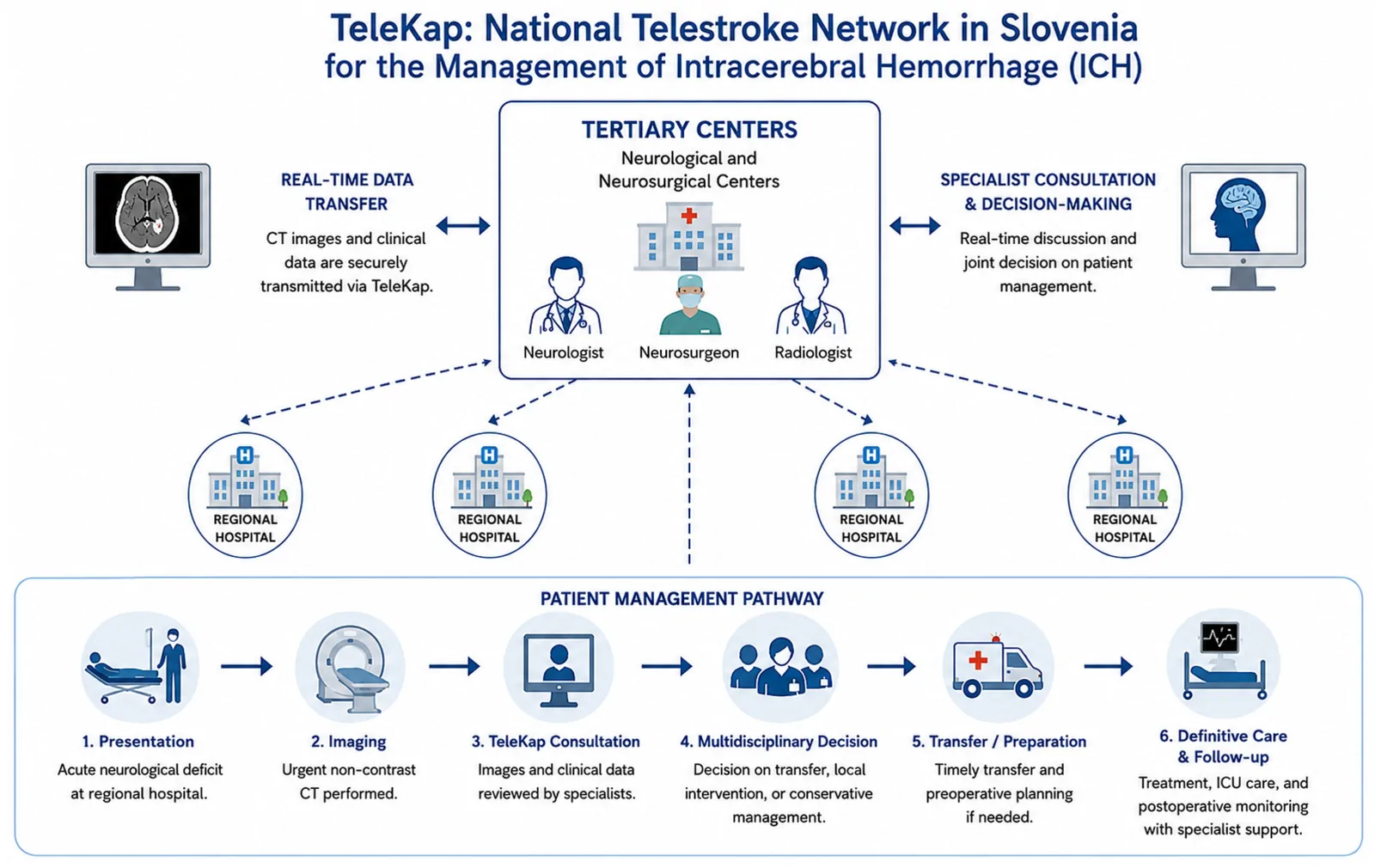
Organisational structure and principal functions of TeleKap in intracerebral haemorrhage (ICH). The network links regional hospitals with tertiary neurological and neurosurgical centres through audiovisual consultation and remote access to clinical and imaging data. The figure depicts the proposed pathway from initial assessment to specialist-supported triage, transfer, local management, or preoperative preparation. These are organisational capabilities; their effect on ICH treatment times and outcomes has not been directly evaluated.

TeleKap, the Slovenian national telestroke network, was introduced in 2014 as a centralised telemedicine platform to enable real-time consultation and coordination among healthcare providers across the country. Its primary goal was to improve access to specialist neurological expertise, particularly in time-sensitive conditions such as AIS. Over time, the infrastructure has also been used to support consultation for complex neurological and neurosurgical emergencies, including ICH; its effectiveness for this indication has not yet been formally quantified. A graphical overview of the TeleKap system in the management of ICH in Slovenia is schematised in Figure 1.

Telestroke was developed to address geographical disparities in specialist stroke care and has improved access to reperfusion treatment in predominantly AIS populations [23,24]. TeleKap represents a nationwide adaptation of this model to Slovenia’s relatively small population and geographically distributed hospital system.

TeleKap operates continuously as a hub-and-spoke network connecting regional hospitals with tertiary neurological and neurosurgical centres. Clinicians communicate by audio or video and share computed tomography (CT) or magnetic resonance imaging (MRI) examinations through a centralised platform. In suspected ICH, remote specialists can integrate haematoma location and extent, intraventricular extension, mass effect, neurological findings, comorbidities, and laboratory results when advising on further imaging, local management, or transfer. Telemedicine-based assessment can achieve diagnostic agreement comparable to bedside consultation, although hands-on examination remains essential [24,25,26,27].

TeleKap functions as a consultation and coordination platform integrated into existing regional and tertiary stroke pathways rather than as a mandatory national stroke registry. Accordingly, this review does not report consultation volumes, treatment or transfer rates, or TeleKap-specific clinical outcomes; these measures require prospective registry-based evaluation.

At an organisational level, TeleKap can provide a common route for specialist consultation and evidence-based recommendations across participating hospitals. This may promote more consistent practice, but depends on reliable connectivity, interoperable imaging systems, trained personnel, and local capacity to implement the advice received [28]. These prerequisites and limitations are considered in Section 6.

## 4. Role of TeleKap in the Management of Intracerebral Haemorrhage

For ICH, TeleKap applies the national network described above to a structured sequence of initial assessment, remote specialist review, management selection, and, when indicated, transfer or preoperative preparation. The following subsections describe the distinct stages of that pathway rather than its presumed clinical benefits.

### 4.1. TeleKap-Supported Initial Assessment and Triage

Patients presenting to regional hospitals with suspected ICH undergo non-contrast brain CT, after which neuroimaging and relevant clinical data are transmitted for specialist review. Neurologists and neurosurgeons integrate radiological findings with neurological status, vital signs, comorbidities, and laboratory results to advise whether additional imaging, local conservative treatment, tertiary transfer, or urgent intervention should be considered [29,30].

### 4.2. TeleKap-Assisted Neurosurgical Decision-Making

Neurosurgical review focuses on features relevant to current ICH guidance, including haematoma volume and location, mass effect, midline shift, intraventricular extension, hydrocephalus, neurological status, and the patient’s overall condition [15,16,17]. Communication can continue if deterioration occurs, allowing the initial recommendation and transfer priority to be reassessed.

### 4.3. TeleKap in Preoperative Planning and Remote Specialist Support

When transfer is planned, advance access to imaging and clinical information permits the receiving centre to assess operative requirements and prepare neurosurgical, anaesthetic, intensive-care, blood-product, and equipment resources before arrival [18]. Whether this shortens time to intervention within TeleKap has not been quantified.

In selected situations where immediate transfer is not feasible, TeleKap also enables remote neurosurgical support for regional hospitals. Depending on local expertise and available resources, specialists may assist with emergency management decisions or provide procedural guidance for life-saving interventions. Although definitive neurosurgical treatment is generally performed at tertiary centres, this capability expands access to specialist expertise during time-critical situations [18,19,20,21].

### 4.4. Standardised TeleKap Workflow for ICH Management

**Figure 2:**
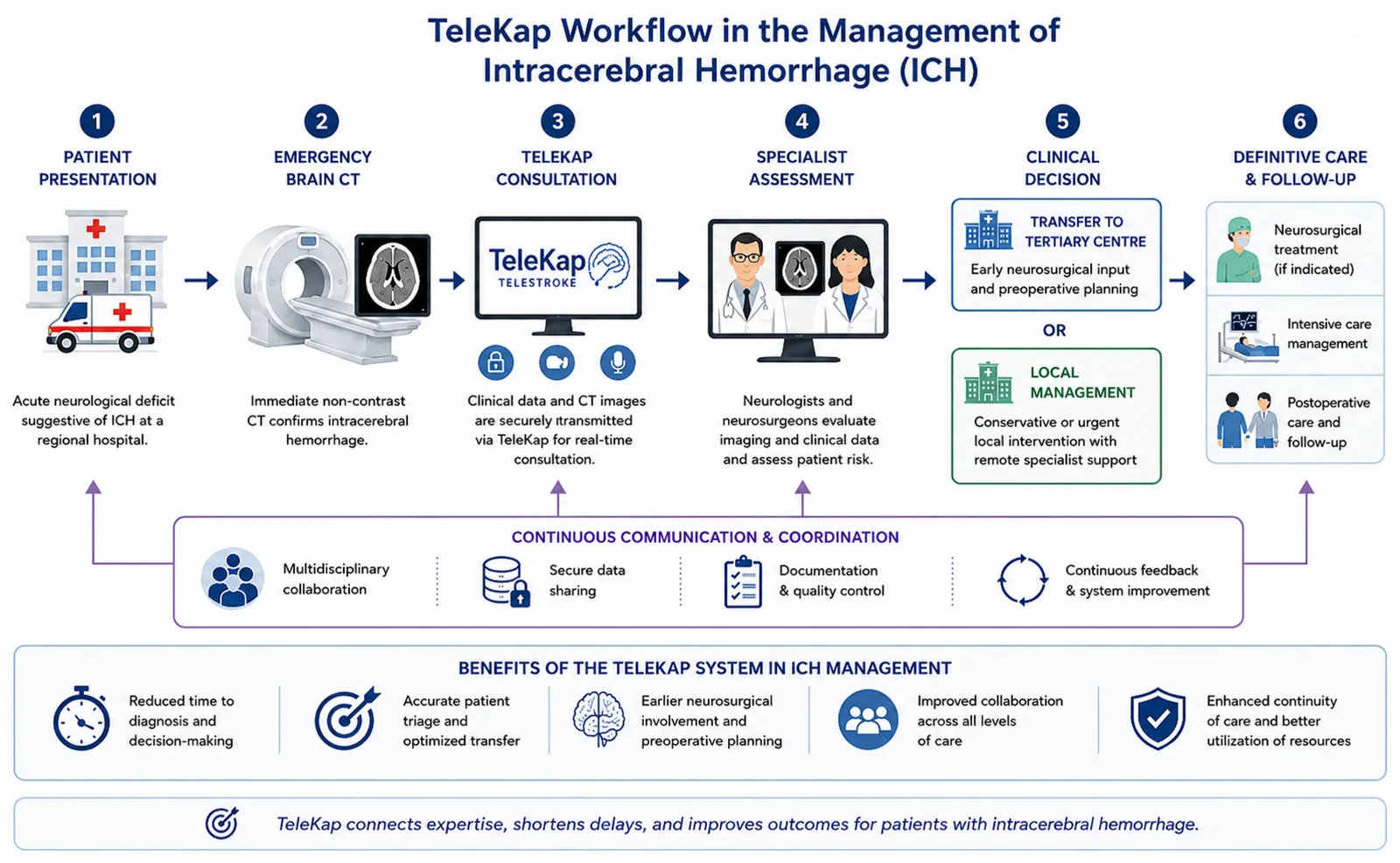
Proposed TeleKap-supported workflow for intracerebral haemorrhage (ICH). After assessment and non-contrast CT at the regional hospital, clinical and imaging data are reviewed remotely by tertiary specialists. The resulting recommendation may be local conservative management, additional investigation, urgent local action when transfer is not feasible, or transfer for neurocritical or neurosurgical care. For transferred patients, the receiving centre may begin preparation before arrival. The workflow depicts intended coordination and does not demonstrate improved ICH outcomes.

Figure 2 integrates these stages into a single proposed workflow, while Table 1 distinguishes the network contribution from the corresponding potential clinical or organisational benefit.

## 5. Potential Organisational Advantages and Current Evidence Gaps

The principal organisational capability of TeleKap is that it provides regional hospitals with access to neurological and neurosurgical input within a nationally coordinated pathway. This capability may improve access to specialist expertise, but it should be distinguished from clinically validated benefit. No TeleKap-specific ICH registry, consecutive cohort, or comparative study has demonstrated shorter treatment times, reduced mortality, improved functional outcomes, or cost-effectiveness. Evidence of telestroke effectiveness is derived predominantly from AIS and cannot be assumed to apply directly to ICH [31,32]. At the system level, TeleKap enables specialist-supported decisions regarding local management and tertiary transfer. Selective transfer could, in principle, reduce avoidable transport and facilitate the allocation of limited neurosurgical resources; however, these represent proposed organisational advantages rather than measured effects in Slovenian patients with ICH [22,33]. Shared protocols and repeated interaction with tertiary specialists also provide opportunities for practice standardisation and professional education. Although these mechanisms may support guideline adherence, knowledge transfer, and confidence among regional teams [22,34,35], their effects have not been formally evaluated within TeleKap.

### Contextual Evidence from European Telestroke Networks

European telestroke services predominantly use regional hub-and-spoke configurations that connect specialist stroke centres with hospitals lacking continuous on-site neurological coverage. In the European Stroke Organisation survey, 25 networks from 10 countries reported a mean of 1.6 hubs and 9 spokes per network; all used two-way audiovisual communication, 24 of 25 primarily supported emergency diagnosis and triage, and the mean proportion of patients receiving reperfusion treatment at spoke hospitals was 12.3% [36]. These data describe network reach and activity rather than intracerebral haemorrhage (ICH)-specific effectiveness. Substantial variation in network size, governance, technology, financing, registry coverage and integration with transfer pathways also limits direct comparison between services [36,37].

Published performance evidence is strongest for acute ischaemic stroke. In the German TEMPiS (The Telemedical Project for Integrative Stroke Care) network, the proportion of patients with ischaemic stroke receiving intravenous thrombolysis increased from 2.6% in 2003 to 15.5% in 2012, while the proportion of patients with stroke or transient ischaemic attack treated in hospitals with telemedicine-supported stroke units increased from 19% to 78% [38]. A comparison of the decentralised TEMPiS model with the centralised Helsinki system further showed that different organisational configurations can achieve substantial population access to thrombolysis, although their treatment rates and delays differed [39]. Decision-analytic studies have also suggested that telestroke can be cost-effective for acute ischaemic stroke, but such estimates depend on model assumptions and cannot be extrapolated directly to ICH or to the Slovenian health system [40].

TeleKap follows the same general hub-and-spoke principle but operates as a nationally coordinated service. Its published evaluation involved one comprehensive stroke centre and 12 partner hospitals and found an association between implementation and increased intravenous thrombolysis use, particularly in hospitals without continuous on-site neurological expertise [32]. These findings provide evidence of service uptake in AIS but do not demonstrate effectiveness in ICH. European telestroke indicators therefore offer contextual benchmarks but cannot be used to infer TeleKap-specific effects on consultation or transfer times, access to neurosurgical intervention, clinical outcomes, or resource utilisation in patients with ICH.

## 6. Challenges and Limitations

Despite the potential organisational advantages of TeleKap in supporting ICH management, several challenges and limitations must be considered. These factors may affect the effectiveness of telemedicine in clinical practice and highlight areas for future improvement and optimization. One of the primary limitations of TeleKap is its reliance on reliable technical infrastructure. The system depends on stable internet connectivity, secure data transmission, and efficient integration of imaging platforms across multiple institutions. Any disruption, such as network failure, slow data transfer, or incompatibility between hospital information systems, can delay image sharing and specialist consultation. In time-critical conditions such as ICH, even brief delays may negatively impact clinical outcomes. Studies on telemedicine in acute stroke care have emphasized that technical reliability is essential for maintaining workflow efficiency and ensuring timely decision-making [41]. Therefore, continuous investment in robust digital infrastructure and system maintenance is crucial for the sustainability of TeleKap. Another important challenge is the variability in local surgical capabilities among regional hospitals. While TeleKap enables remote neurosurgical consultation, the ability to perform emergency procedures locally depends on the availability of trained personnel, appropriate equipment, and institutional experience. Not all regional centres are equipped to perform interventions such as external ventricular drainage or decompressive craniectomy. Consequently, patients in certain regions may still require urgent transfer to tertiary centres, potentially causing delays. This variability can create disparities in the level of care delivered locally, despite telemedicine support. Previous studies have highlighted that telemedicine is most effective when combined with adequate local resources and clearly defined treatment protocols [42]. The need for continuous training and education of regional healthcare teams represents another key limitation.

Effective use of the TeleKap system requires not only technical proficiency but also clinical competence in recognizing ICH, performing initial stabilisation, and implementing specialist recommendations. Regional clinicians must be familiar with telemedicine workflows, imaging protocols, and communication procedures. In addition, they must be capable of managing acute neurological emergencies until definitive care is provided. Ongoing education, simulation training, and regular system use are essential to maintain these competencies. Telemedicine has been shown to enhance knowledge transfer and clinical skills; however, its effectiveness depends on active engagement and training of participating teams [42,43].

A further limitation is the risk of delays or disruptions in cases of system failure. Although telemedicine systems are designed to improve efficiency, they also introduce a dependency on technology that may occasionally fail. Technical issues such as software malfunctions, server downtime, or cybersecurity concerns can temporarily interrupt communication between centres. In such situations, clinicians must revert to traditional methods of consultation, such as telephone communication or manual transfer of imaging data, which may be less efficient. Contingency protocols are therefore essential to ensure continuity of care in the event of system failure. The importance of backup systems and redundancy has been emphasised in studies evaluating telemedicine networks [41].

In addition to technical and logistical challenges, there are also limitations related to remote clinical assessment. While TeleKap provides access to imaging and clinical data, it cannot fully replace direct bedside evaluation by a specialist. Certain aspects of neurological examination, such as subtle changes in mental status or evolving focal deficits, may be more difficult to assess remotely. Although video communication can partially mitigate this limitation, it may not always be available or sufficient in all clinical situations. As a result, telemedicine should be viewed as a complementary tool rather than a substitute for in-person care. Another consideration is the potential for medico-legal and organisational challenges. The use of telemedicine involves multiple institutions and healthcare providers, raising questions about responsibility, documentation, and data protection. Clear protocols and legal frameworks are necessary to define roles, ensure patient confidentiality, and standardise documentation practices. These issues have been recognized as important barriers to the widespread implementation of telemedicine systems and require ongoing attention at both institutional and national levels [44]. Furthermore, while TeleKap may improve access to expertise, there may be variability in decision-making among specialists, particularly in complex cases where evidence is limited or controversial [45]. Although telemedicine facilitates consultation, it does not eliminate the inherent uncertainty in certain clinical decisions. This underscores the importance of standardised guidelines and multidisciplinary discussion in ensuring consistent, evidence-based care.

Taken together, these limitations indicate that the sustainability of TeleKap depends on reliable technical infrastructure, clearly defined contingency pathways, adequately trained regional teams, appropriate local resources, and transparent governance arrangements. Its principal organisational advantages and practical limitations are summarised in Table 2.

## 7. Future Perspectives

Future development of TeleKap should be considered in relation to its current capabilities and the practical needs of the Slovenian stroke system. Although digital technologies are increasingly being investigated in telestroke and neuroemergency care [24,44,45,46], advanced artificial intelligence (AI), machine-learning decision support, automated haematoma volumetry, wearable monitoring, mobile stroke units and Tele-ICU technologies are not currently integrated into TeleKap to any meaningful degree. These technologies should therefore be presented as possible future developments rather than components of established TeleKap practice. The network’s immediate priorities are more fundamental and include prospective data collection, standardisation of consultation and transfer protocols, interoperability between participating hospitals, and systematic evaluation of clinical and organisational performance.

The establishment of a mandatory national stroke registry, or a dedicated prospective TeleKap registry linked with hospital outcome data, would represent the most important next step. Such a system should record stroke subtype, consultation indication, relevant time intervals, treatment recommendations, interhospital transfers, neurosurgical or endovascular procedures, complications, mortality and functional outcomes. For patients with ICH, particularly relevant indicators would include consultation-response time, time to anticoagulant reversal, transfer-decision time, time to neurosurgical assessment or intervention, the proportion of potentially avoidable transfers, and outcomes according to local versus tertiary-centre management. This evidence base is necessary before the effectiveness and cost-effectiveness of TeleKap can be assessed reliably.

Among emerging technologies, AI-supported analysis of CT and MRI examinations represents the most realistic medium-term direction for TeleKap. Validated software could assist with the detection and quantification of early ischaemic changes, large-vessel occlusion and perfusion abnormalities. In haemorrhagic stroke, it could support ICH detection, automated haematoma volumetry, identification of intraventricular extension and imaging markers associated with haematoma expansion, and comparison of haematoma volume on serial examinations [18,44,45,46,47]. Integration of these outputs into the existing image-sharing pathway could facilitate more standardised communication and triage between regional and tertiary centres. Nevertheless, AI-supported imaging should complement rather than replace specialist interpretation, and its introduction would require local validation, technical interoperability, appropriate data governance and prospective assessment of its effect on workflow and patient outcomes.

Other technologies described in the broader telemedicine literature—including Tele-ICU support, wearable physiological monitoring, and mobile stroke units—are not currently implemented within TeleKap and should be regarded as longer-term possibilities rather than immediate priorities [18,48,49,50]. Their potential adoption would require separate evaluation of feasibility, infrastructure, staffing, governance, and cost within the Slovenian healthcare system. Accordingly, rather than pursuing the broad and immediate adoption of multiple emerging technologies, TeleKap should develop stepwise as a measurable national service: registry-based quality monitoring, protocol standardisation, and interoperability should precede the introduction of selected innovations—most plausibly AI-supported CT and MRI analysis—which should undergo local validation and prospective evaluation before routine implementation [48,49,50]. Accordingly, TeleKap should develop stepwise: registry-based quality monitoring, protocol standardisation, and improved interoperability represent immediate priorities; locally validated AI-supported imaging may constitute a realistic medium-term development; and resource-intensive technologies such as Tele-ICU, wearable monitoring, and mobile stroke units should remain longer-term possibilities requiring separate feasibility and economic evaluation. The proposed priorities for the stepwise development of TeleKap are summarised in Table 3, distinguishing immediate organisational and measurement needs from medium-term AI-supported imaging and longer-term technology-dependent developments.

## 8. Discussion

This review provides a focused description of how the Slovenian TeleKap network is used operationally in ICH. Its contribution lies in examining a national implementation within a specific healthcare context rather than in proposing a novel telestroke or teleneurosurgical model. Comparable organisational elements—including hub-and-spoke consultation, remote neuroimaging review, transfer coordination, and access to neurosurgical advice—are well represented in the broader telemedicine literature [15,16,17,18,19,20,21,22]. The present review neither provides original ICH outcome data nor demonstrates the clinical effectiveness of TeleKap. Very elderly patients (≥85 years) represent a particularly important subgroup of patients with intracerebral haemorrhage because of their distinct demographic characteristics, underlying aetiologies, and clinical outcomes. Earlier clinical studies demonstrated that these patients more frequently present with impaired consciousness, extensive neurological deficits, and higher in-hospital mortality than younger patients [51]. More recent evidence, however, suggests that although the oldest-old often present with larger haematoma volumes, advanced age itself is not an independent predictor of mortality after adjustment for haemorrhage characteristics and clinical severity. These findings emphasize that treatment decisions should be individualized rather than based solely on chronological age [52]. In this context, the TeleKap system may be particularly valuable by enabling rapid multidisciplinary assessment of imaging findings, neurological status, premorbid functional condition, and comorbidities, thereby supporting individualized triage and management decisions in very elderly patients. The present review did not specifically analyse patients aged ≥85 years as a separate subgroup because its primary objective was to describe the role of the Slovenian TeleKap network in the organization and management of spontaneous intracerebral haemorrhage rather than to evaluate outcomes in specific patient populations. Nevertheless, we agree that this subgroup is clinically important and have incorporated a discussion of the available evidence and its implications for telemedicine-supported patient triage.

The clinical rationale for extending telestroke infrastructure to ICH is strong: neurological deterioration and haematoma expansion often occur early, while decisions about surgery remain time-sensitive and highly dependent on patient selection [24,46,49,50]. Nevertheless, organisational plausibility is not evidence of effectiveness. Studies of TeleKap should therefore compare process measures and outcomes against clearly defined conventional referral pathways rather than infer benefit from its technical capabilities.

TeleKap may be informative for health systems that similarly combine centralised neurosurgical services with uneven regional specialist availability. Its proposed value lies in applying established telemedicine principles to multidisciplinary ICH pathways, including blood-pressure management, anticoagulant reversal, neurocritical care, and selection for surgery [1,23,53]. However, the extent to which observations from Slovenia add to, or differ from, experience with other telestroke and teleneurosurgical networks cannot be determined without comparative data. Claims regarding fewer transfers or more efficient resource use remain hypotheses requiring evaluation [25,26,27,54,55].

The Slovenian healthcare system has contextual features—including a relatively small population, nationwide network coverage, and centralised specialist services—that shape how TeleKap operates. These features should not be interpreted as evidence that the underlying organisational model is unique or readily generalisable. Its core components overlap with previously described telestroke and teleneurosurgical systems, while differences in geography, staffing, referral pathways, financing, and digital infrastructure may limit transferability. TeleKap should therefore be viewed as one national implementation among several possible organisational approaches rather than as a new paradigm for international neuroemergency care.

TeleKap should therefore be regarded as a context-dependent national implementation of established telemedicine principles rather than as a novel or readily transferable paradigm. Its relevance to other healthcare systems will depend on differences in geography, specialist availability, referral pathways, financing, and digital infrastructure.

## 9. Conclusions

TeleKap provides a nationwide organisational framework through which regional clinicians can obtain neurological and neurosurgical support for patients with ICH within Slovenia’s centralised specialist healthcare system. This review describes its potential contribution to specialist assessment, triage, and interhospital coordination but does not establish its clinical effectiveness, cost-effectiveness, or transferability. Further development should prioritise registry-based quality monitoring, standardised protocols, and interoperability, with locally validated AI-supported imaging considered as a subsequent medium-term step.

## Figures and Tables

**Table 1:** Organisational functions of TeleKap across the proposed ICH management pathway and their potential implications. The second column describes functions available through the network, whereas the third presents hypothesised organisational or clinical implications that have not been validated as effects on treatment processes or outcomes in patients with ICH assessed through TeleKap.

Stage of ICH Management	Organisational Function	Potential Implication—Not Clinically Validated
Initial assessment	Immediate transmission of CT images and clinical information from regional hospitals to tertiary centres	May facilitate earlier specialist involvement
Diagnosis	Real-time remote review of neuroimaging by neurologists and neurosurgeons	May support specialist characterisation of ICH and risk stratification
Patient triage	Multidisciplinary specialist consultation	May support better-informed selection for local management or tertiary transfer
Neurosurgical decision-making	Remote assessment of haematoma size, location, mass effect, intraventricular haemorrhage, and neurological status	May assist identification of patients for whom neurosurgical intervention is considered
Interhospital transfer	Early communication between referring and receiving hospitals	May facilitate coordination of patient transport
Preoperative planning	Advance review of imaging and clinical data before patient arrival	May permit advance mobilisation of surgical teams and preparation of operating theatre and intensive care resources
Local management	Remote specialist guidance for conservative treatment or emergency procedures when transfer is not immediately feasible	May extend access to specialist guidance when immediate transfer is not feasible
Definitive treatment	Continuous communication between multidisciplinary teams	May support coordination between multidisciplinary teams
Postoperative care	Coordination between tertiary and regional centres during recovery and follow-up	May support continuity between tertiary and regional care
Quality improvement	Standardised protocols, multidisciplinary communication, and continuous feedback	May promote practice consistency, coordinated resource use, and guideline adherence

CT—computed tomography, ICH—intracerebral haemorrhage. The listed implications represent plausible consequences of the organisational functions of TeleKap; none has been directly validated in a TeleKap-specific ICH cohort.

**Table 2:** Principal organisational advantages and practical limitations of TeleKap in the management of intracerebral haemorrhage.

Aspect	Advantages	Potential Limitations
Diagnosis	Rapid image sharing and specialist interpretation	Dependence on image quality and network connectivity
Consultation	Immediate neurological and neurosurgical expertise	Limited bedside examination
Triage	Specialist-supported referral decisions	Variability in local clinical resources
Surgery	Early preoperative planning	Surgical capability differs among regional hospitals
Collaboration	Continuous multidisciplinary communication	Requires coordinated workflows
Infrastructure	Nationwide coordinated network	Vulnerable to technical failures
Education	Knowledge transfer and support for regional clinicians	Requires regular training and competency maintenance

**Table 3:** Prioritised future development of the TeleKap network. Registry-based quality monitoring, protocol standardisation, and interoperability represent immediate priorities. Locally validated AI-supported imaging constitutes a possible medium-term development, whereas Tele-ICU support, wearable monitoring, and mobile stroke units remain longer-term possibilities requiring separate feasibility and economic evaluation.

Priority	Development Category	Principal Objective
Immediate	National registry and quality monitoring	Systematic collection of consultation, treatment, transfer, safety, and outcome indicators to enable evaluation of TeleKap performance
Immediate	Interoperability and standardised protocols	Reliable exchange of clinical and imaging data and consistent consultation, triage, transfer, and contingency procedures across participating hospitals
Medium term	Validated AI-supported imaging	Support for ICH detection, haematoma volumetry, identification of intraventricular extension and high-risk imaging features, and more efficient image routing and specialist notification
Longer term	Tele-ICU, wearable monitoring, and mobile stroke units	Potential extension of monitoring and specialist support beyond the current TeleKap pathway, subject to separate feasibility, infrastructure, staffing, governance, and economic evaluation

AI—artificial intelligence, ICH—intracerebral haemorrhage, ICU—intensive care unit.

## Data Availability

No new data were created or analyzed in this study. Data sharing is not applicable to this article.

## Abbreviations

The following abbreviations are used in this manuscript:

AI	Artificial intelligence
AIS	Acute ischaemic stroke
CT	Computed tomography
ICH	Intracerebral haemorrhage
MRI	Magnetic resonance imaging
Tele-ICU	Tele-intensive care
TEMPiS	The Telemedical Project for Integrative Stroke Care
